# From animal models to metabolic interventions: Lessons from the Wobbler mouse

**DOI:** 10.4103/NRR.NRR-D-25-01456

**Published:** 2026-01-27

**Authors:** Veronika Matschke

**Affiliations:** Department of Cytology, Institute of Anatomy, Medical Faculty, Ruhr-University Bochum, Bochum, Germany; International Graduate School of Neuroscience (IGSN), Ruhr-University Bochum, Bochum, Germany

Motor neuron diseases such as amyotrophic lateral sclerosis (ALS) remain largely incurable, with limited therapeutic options and only modest clinical benefits from currently approved drugs. Experimental models have been instrumental in shaping our understanding of disease mechanisms, yet translation into effective therapies has been challenging. Among these models, the Wobbler mouse, although less frequently employed than superoxide dismutase 1 (SOD1) transgenics, offers unique insights into motor neuron degeneration driven by disrupted intracellular trafficking and metabolic stress.

Our recent work with caffeine as a metabolic modulator highlights the potential of targeting interconnected pathways such as redox balance and nicotinamide adenine dinucleotide (NAD^+^) metabolism to delay neuronal loss (Epplen et al., 2025). NAD^+^ availability governs sirtuin activity and mitochondrial DNA repair, and impaired antioxidant-based defenses further tip the balance toward oxidative damage (Wunsch et al., 2023). These pathways converge on processes central to neuronal vulnerability. Thus, metabolic interventions should be viewed not as supportive strategies but as central components in reshaping how we approach neurodegeneration. This Perspective argues that the Wobbler model deserves renewed attention as a platform for exploring such vulnerabilities. Lessons from this system may help uncover interventions that transcend ALS and inform broader strategies for neurodegenerative disease treatment.

**Amyotrophic lateral sclerosis — bridging the gap between models and the clinic:** ALS is a devastating neurodegenerative disorder characterized by progressive loss of upper and lower motor neurons. Despite decades of research and the development of various experimental models, therapeutic progress remains limited. Current treatments such as riluzole and edaravone extend survival only by months. More recently, the antisense oligonucleotide tofersen has been approved for patients carrying SOD1 mutations, yet its benefits remain restricted to a small genetic subset and rely largely on surrogate biomarkers rather than robust clinical endpoints (Ansari et al., 2024). Together, these limitations underscore the urgent need for new mechanistic insights and broadly applicable intervention strategies.

Animal models have been central to ALS research, but the translation of preclinical findings into clinical success has been notoriously difficult. A major reason lies in the narrow reliance on a few dominant models, most notably transgenic SOD1 mice, that, while invaluable, represent only a fraction of the heterogeneity of the disease. Other frequently used systems, such as TAR DNA-binding protein 43 (TDP-43), fused in sarcoma (FUS), or C9orf72 repeat-expansion models, primarily recapitulate genetic or protein-driven mechanisms and rely on forced expression of human mutant proteins. These approaches have generated key mechanistic insights, yet they capture metabolic imbalance, vesicle-trafficking defects, and redox vulnerability only incompletely, as these features often arise downstream rather than as initiating events.

ALS is increasingly recognized as a multifactorial syndrome rather than a uniform entity, with contributions from oxidative stress, impaired axonal transport, metabolic imbalance, and neuroinflammation. In addition, defects in autophagy, endoplasmic reticulum stress, and altered lipid metabolism contribute to neuronal decline. Hyperexcitability mediated by glutamatergic overdrive and impaired clearance of extracellular glutamate via astrocytic EAAT2 transporters further accelerates excitotoxic cascades (Mejzini et al., 2019). Yet many of these features are underrepresented in traditional models, limiting the translational scope of experimental interventions.

Bridging this gap requires a critical reassessment of the systems we use to study ALS. Beyond diversifying the experimental toolbox, it also demands a reframing of therapeutic priorities: shifting from late-stage symptom-oriented strategies toward interventions that stabilize metabolism and cellular homeostasis at earlier disease stages.

**Wobbler mouse as an underestimated but valuable model:** Among the available animal models of ALS, the Wobbler mouse holds a distinct yet underappreciated position. The spontaneous mutation in the *Vps54* gene leads to impaired vesicle trafficking and progressive motor neuron degeneration, producing a phenotype that shares key pathological features with human ALS, including muscle weakness, axonal degeneration, gliosis, and mitochondrial dysfunction (Mejzini et al., 2019). Clinically, Wobbler mice progress through three stages: a pre-symptomatic phase during the first three postnatal weeks in which they are indistinguishable from wild-type littermates, a rapidly progressive phase beginning around p20 characterized by emerging forelimb weakness and motor neuron degeneration, and a later plateau phase from approximately p40 onward during which symptoms stabilize but remain pronounced. Unlike SOD1 models, which represent a small subset of familial ALS, the Wobbler mouse reflects aspects more relevant to sporadic disease, making it a valuable complement in the experimental landscape.

What distinguishes the Wobbler model is its pronounced involvement of metabolic stress. Motor neurons in Wobbler mice display early signs of oxidative stress and disrupted mitochondrial homeostasis (Stein et al., 2021; Junghans et al., 2022; Wunsch et al., 2023), alterations that align with systemic metabolic dysfunction increasingly recognized in ALS patients (Maksimovic et al., 2023). In addition to altered mitochondrial network morphology and ROS-driven mitochondrial fragmentation, early impairments in complex I activity and reduced respiratory capacity have been reported in the spinal cord and motor cortex of Wobbler mice (Dave et al., 2003). Comparable mitochondrial abnormalities, including disrupted transport, loss of membrane potential, and increased fragmentation, have been reported in SOD1 and TDP-43 transgenic models, although these changes typically appear later or as downstream consequences of protein aggregation (Mattiazzi et al., 2002; Magrané et al., 2009; Wang et al., 2013). In parallel, deficits in vesicular trafficking impair delivery of survival factors and mitochondrial components to distal axons, compounding energy failure (Kürten et al., 2023). Abnormal activation of microglia and astrocytes enhances neuroinflammation through nuclear factor κB signaling, leading to elevated cytokine and chemokine release (Cihankaya et al., 2024). Additionally, the Wobbler model has revealed vulnerabilities in the glutathione system. Reduced glutathione availability increases oxidative DNA damage, particularly double-strand breaks, highlighting impaired antioxidant defenses as a critical node of pathology (Wunsch et al., 2023). Restoration of glutathione precursors or mitochondrial-targeted antioxidants can attenuate such damage, underscoring the therapeutic potential of strengthening endogenous antioxidant systems (Junghans et al., 2022).

Taken together, these features make the Wobbler mouse particularly suitable for testing interventions that target metabolic and redox pathways. Yet despite these advantages, the model has been relatively neglected, overshadowed by the dominance of transgenic systems. Its unique pathophysiological profile offers an opportunity to interrogate mechanisms beyond protein misfolding, expanding our understanding of ALS as a disorder of interconnected metabolic and cellular networks. Harnessing the potential of this model may help identify therapeutic strategies with higher translational relevance.

**Metabolic interventions as proof-of-concept — the case of caffeine:** Over the past decade, evidence has converged on the central role of metabolic dysregulation in ALS (Maksimovic et al., 2023). Disturbances in mitochondrial respiration, oxidative stress, NAD^+^ homeostasis, iron handling, and glutathione metabolism all contribute to motor neuron vulnerability (Mejzini et al., 2019). This recognition has shifted the therapeutic focus toward strategies that enhance bioenergetic resilience and redox balance.

Caffeine, one of the most widely consumed psychoactive substances, has emerged as a potential neuroprotective agent. Epidemiological data suggest inverse associations between caffeine intake and the risk of neurodegenerative diseases such as Parkinson’s disease and Alzheimer’s disease. Preclinical studies demonstrate that caffeine exerts antioxidative, antiapoptotic, and anti-inflammatory effects. Mechanistically, caffeine acts as a nonselective adenosine receptor antagonist, attenuating excitotoxic signaling, while also influencing calcium handling, mitochondrial efficiency, and NAD^+^ dependent pathways. A1 receptor blockade reduces neuronal hyperexcitability, while A2A antagonism attenuates microglial activation and suppresses nuclear factor κB dependent transcription of pro-inflammatory genes. By improving mitochondrial coupling efficiency and limiting excessive calcium influx, caffeine prevents opening of the mitochondrial permeability transition pore, thereby preserving adenosine triphosphate generation and preventing apoptotic cascades. Caffeine also modulates cAMP-PKA-pathways, which intersect with CREB-mediated transcription of genes important for neuronal survival (Rei et al., 2022; Epplen et al., 2025). In our recent study, caffeine administration in the Wobbler mouse reduced reactive oxygen species and delayed motor neuron degeneration. Notably, studies in SOD1 mice have reported model-dependent effects of chronic caffeine intake, including altered survival and adenosine-receptor signaling, underscoring that the impact of caffeine varies across ALS models and is shaped by their distinct metabolic vulnerabilities (Rei et al., 2022). Beyond antioxidative activity, caffeine suppressed inflammasome activation, decreased interleukin (IL)-1β and IL-18 levels, and modulated microglial activation, highlighting anti-inflammatory effects that are increasingly recognized as central to ALS pathology (Epplen et al., 2025). At the immunological level, inhibition of NLRP3 inflammasome assembly reduces caspase 1 activation and limits downstream IL-1β and IL-18 release (Cihankaya et al., 2024). In parallel, enhanced NAD^+^ turnover may reinforce sirtuin mediated deacetylation of peroxisome proliferator-activated receptor gamma coactivator 1α, a master regulator of mitochondrial biogenesis.

**Figure 1:**
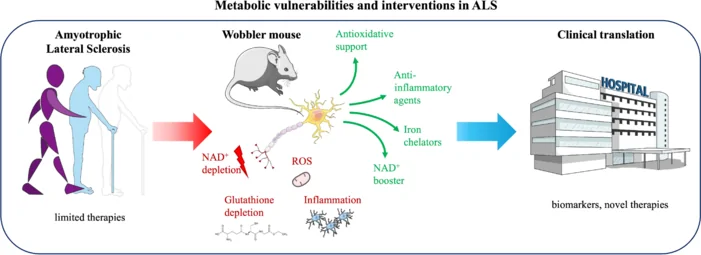
Schematic overview of metabolic vulnerabilities in ALS and therapeutic opportunities revealed by the Wobbler mouse. The figure summarizes key metabolic disturbances characteristic of the Wobbler model, including early NAD^+^ depletion, mitochondrial ROS accumulation, glutathione deficiency, and inflammatory activation, all of which contribute to motor neuron degeneration. The schematic also highlights representative intervention strategies: antioxidative support, anti-inflammatory agents, iron chelators, and NAD^+^-boosting approaches, illustrating how targeting these interconnected pathways may guide the development of metabolic therapies and support translation toward clinical application. Adapted from Servier Medical Art (https://smart.servier.com), licensed under CC BY 4.0 (https://creativecommons.org/licenses/by/4.0/). ALS: Amyotrophic lateral sclerosis, NAD^+^: nicotinamide adenine dinucleotide, ROS: reactive oxygen species.

Beyond caffeine, the Wobbler model has recently been used to test other metabolically oriented interventions. Inhibition of sphingolipid synthesis with myriocin, a serine palmitoyltransferase inhibitor, corrected the accumulation of cytotoxic sphingoid bases, reduced astrogliosis, improved grip strength, and extended survival in Wobbler mice, directly linking lipid metabolism to motor neuron integrity in this model (Petit et al., 2020). In parallel, metabolic approaches in other ALS models, such as provision of alternative fuels or mitochondrial-targeted antioxidants in SOD1 mice, have been shown to protect motor neurons and delay symptom onset (Maksimovic et al., 2023). Taken together, metabolic interventions in the Wobbler mouse and in other ALS models, including alternative fuel supplementation or mitochondrial-targeted antioxidants, illustrate that modifying energy and lipid homeostasis can influence disease progression. Combined with emerging evidence on glutathione metabolism, redox imbalance, iron handling and inflammation, these findings suggest that accessible, well-tolerated compounds may serve not only as symptomatic agents but as entry points to broader therapeutic principles. Caffeine fits into this conceptual framework by demonstrating that targeting NAD^+^ metabolism, oxidative stress, and inflammatory signaling can alter disease trajectory and motivate the exploration of more potent metabolic modulators. A graphical summary of these interconnected metabolic vulnerabilities and potential intervention points is provided in **Figure 1**.

**Outlook and future directions:** The lessons from the Wobbler mouse highlight a broader principle: ALS is not only a disease of motor neurons, but a disorder of systemic homeostasis. Metabolic imbalance, oxidative stress, glutathione depletion, iron dysregulation, and chronic inflammation converge to create a permissive environment for degeneration. Interventions that stabilize these processes may hold genuine therapeutic promise. While caffeine provides an illustrative proof of concept, its modest potency and pleiotropic nature point to the need for more specific modulators. Strengthening endogenous antioxidant support from astrocytes through pathways such as nuclear factor erythroid 2-related factor 2 may also be a productive route.

Future research should pursue two parallel paths: mechanistic refinement and clinical translation. Refining animal models such as the Wobbler mouse can deepen our understanding of how metabolic stress drives neurodegeneration and support rigorous preclinical evaluation of agents that modulate NAD^+^ metabolism, mitochondrial dynamics, iron handling or glutathione availability. Translational studies must determine whether these interventions are feasible and effective in ALS patients. Given the heterogeneity of ALS, biomarkers of oxidative stress, iron homeostasis, glutathione metabolism and mitochondrial function could help identify patient subgroups defined by distinct metabolic vulnerabilities. Such stratification would not only support therapeutic monitoring but also guide the selection of individuals most likely to benefit from specific metabolic interventions. The integration of multi-omics approaches including metabolomics, transcriptomics and proteomics may help identify metabolic bottlenecks that define patient subgroups responsive to targeted therapies. Moreover, combination strategies that pair metabolic interventions with anti-excitotoxic or anti-inflammatory drugs may provide synergistic benefits.

More broadly, the concept of targeting metabolism resonates across neurodegenerative diseases. Because mitochondrial dysfunction, glutathione depletion, and chronic inflammation are shared hallmarks, insights gained from metabolic interventions in ALS may extend to Alzheimer’s disease, where impaired glucose utilization and oxidative stress dominate, and to Parkinson’s disease, where mitochondrial complex I inhibition and alpha-synuclein aggregation converge. The Wobbler mouse exemplifies how non-classical models can guide cross-disease therapeutic innovation. By bridging the gap between models and clinical translation, metabolic interventions hold the potential to redefine the therapeutic landscape of neurodegeneration.
